# The Presence of Communicating Arteries in the Circle of Willis Is Associated with Higher Rate of Functional Recovery after Anterior Circulation Ischemic Stroke

**DOI:** 10.3390/biomedicines11113008

**Published:** 2023-11-09

**Authors:** Sara Sablić, Krešimir Dolić, Ivan Kraljević, Danijela Budimir Mršić, Mate Čičmir-Vestić, Benjamin Benzon, Sanja Lovrić Kojundžić, Maja Marinović Guić

**Affiliations:** 1 Clinical Department of Diagnostic and Interventional Radiology, University Hospital of Split, 21000 Split, Croatia; sarasablic@gmail.com (S.S.);; 2 University of Split School of Medicine, 21000 Split, Croatia; 3 University Department of Health Studies of the University of Split, 21000 Split, Croatia; 4 Department of Neurology, University Hospital of Split, 21000 Split, Croatia

**Keywords:** ischemic stroke, mechanical thrombectomy, circle of Willis, endovascular treatment

## Abstract

Acute ischemic stroke (AIS) is the world’s second leading cause of mortality. An established method for treating stroke patients in acute settings is endovascular therapy (EVT). However, the correlation of the successful endovascular treatment of AIS with the presence of communicating arteries in the circle of Willis needs to be proven. Our study examined clinical and radiological data of 158 consecutive patients treated with mechanical thrombectomy (MT) at our comprehensive stroke center. We analyzed their CT angiograms and digital subtraction angiography (DSA) to assess anatomical variants of Willis’ circle and formed two groups—collateral-negative and collateral-positive group. The first group included patients with aplasia of both anterior (ACoA) and posterior communicating Artery (PCoA). The second group included patients that have at least one communicating artery (either anterior or posterior). We evaluated their reperfusion outcomes and functional recovery three months later. Our results showed that patients with communicating arteries had smaller areas of infarction on post-interventional CT and higher rates of functional recovery (Modified Rankin Score). The ACoA had a higher impact on early and late outcomes, confirmed by lower control CT scores and more favorable functional recovery. Therefore, anatomic variants of Willis’ circle should be considered as a significant prognostic factor in AIS.

## 1. Introduction

Acute ischemic stroke (AIS) is the second leading cause of mortality and a significant contributor to death and disability in the world [1,2]. Several comorbidities have been identified as risk factors for anterior circulation ischemic stroke, including high body mass index (BMI > 25), the atherosclerosis of extracranial and intracranial carotid arteries, atrial fibrillation, smoking, and alcohol consumption [3,4,5,6,7]. Endovascular therapy (EVT) with an aspiration catheter and stent retrievers, with or without the intravenous administration of thrombolysis, is the main treatment option for AIS caused by large vessel occlusion (LVO) within a 24 h window [8]. When a cerebral artery occludes (due to thromboembolism, hemodynamic impairment, or a combination of these causes), an alternative arterial pathway is triggered for perfusion to the ischemic brain areas. A pressure gradient that directs blood flow in the various segments of the circle of Willis (CoW), supplying blood to the cerebral arteries on the side of the occlusion, is created at the location of the occluded artery by a decrease in arterial blood pressure in all ipsilateral cerebral arteries [9]. Early activation primarily involves CoW arterial segments, which are considered primary collaterals, with the late recruitment of the ophthalmic artery and leptomeningeal arteries, which are considered secondary collaterals [10,11,12,13]. In the case of pre-Willis carotid artery occlusion (anterior circulation), which includes the regions of the anterior and middle cerebral arteries as well as the distal internal carotid artery (tandem or T-occlusion), blood flow in the proximal anterior cerebral artery (ACA) via the ophthalmic artery and the anterior communicating artery (ACoA) compensates in the event of an anterior circulation stroke. The posterior communicating arteries can provide collateral blood flow in either direction to the anterior and posterior circulations [9,10,11,12]. During basilar artery occlusion, posterior communicating arteries (PCoAs) reverse blood flow through the basilar bifurcation, Posterior Cerebral Arteries (PCAs), and Superior Cerebellar Arteries (SCAs) [14]. In the case of post-Willis occlusion, the decrease in hemodynamic resistance imposes a rerouting of the blood flow into the leptomeningeal arterial anastomoses in the watershed zone involving various cerebral arteries [9]. A study based on experimental animals showed that the occlusion of the internal carotid artery (ICA) leads to the dilatation of the leptomeningeal collaterals in only 12 s, thus demonstrating the fast recruiting response of vasoconstricted arteries [15]. The most common vascular variants, according to postmortem and imaging studies in humans, were the agenesis or hypoplasia of the ACA, the unilateral fetal type of the PCA, and the complete CoW, which only occurs in a minority of patients [16,17,18,19].

There are several studies that correlated anatomical variants of the Willis circle with outcomes after stroke, using different classifications for anatomical variants [20,21,22,23,24], and their results remained somewhat inconclusive. We hypothesized that the presence of ACoA or PCoA affects functional outcomes after mechanical thrombectomy.

## 2. Materials and Methods

### 2.1. Study Design and Participants

This study complied with the ethical guidelines of the 1975 Declaration of Helsinki and was approved by the institutional ethics committee at the University Hospital in Split, Croatia. Informed consent was waived because of the retrospective nature of the study. We analyzed patients with acute ischemic stroke of the anterior circulation who met the criteria for EVT from 1 January 2021 to 1 July 2023, at the University Hospital in Split, Croatia. The criteria for EVT in anterior circulation stroke were the onset of symptoms less than 6 h before presentation to the Neurology Emergency Department, radiological confirmation of the occlusion, and Modified Rankin Scale Score (mRS) before the occurrence of stroke 0–2. We excluded patients with large vessel occlusion due to carotid artery dissection, patients with ischemic stroke in the posterior circulation (vertebral or basilar artery occlusion), and patients with missing radiological data. Of the 230 mechanical thrombectomies (MT) performed at our comprehensive stroke center during this period, 158 patients met our inclusion criteria. Patient demographics (age, gender, stroke risk factors, comorbidities, and anticoagulant treatment) and clinical data (stroke location, MT technique used, Modified Rankin Scale Score, and National Institutes of Health Stroke Scale (NIHSS) on admission) were collected from the hospital information system (HIS) as well as systolic blood pressure (SBP) upon arrival with a cut-off value of 140 mmHg.

### 2.2. Imaging Data and Analysis

**Figure 1:**
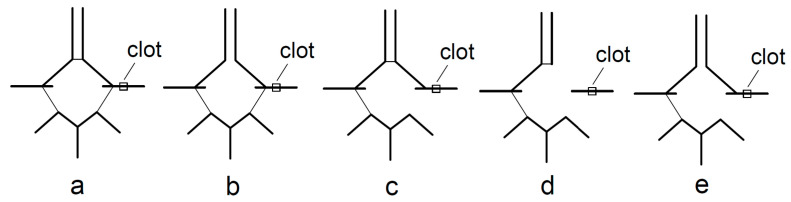
Collateral-positive (**a**–**c**) and collateral-negative group (**d**,**e**): **a**—both ACoA and PCoA are present; **b**—absence of ACoA but presence of PCoA on the side of the clot; **c**—absence of PCoA but presence of ACoA on the side of clot; **d**—presence of ACoA but absence of A1 and absence of PCoA on the side of the clot; **e**—absence of both ACoA and PCoA on the side of the clot.

Before EVT, all patients underwent a stroke protocol that included emergency non-enhanced brain CT, cerebral CT angiography (CTA), and CT perfusion (CTP) on a 128-slice MSCT Somatom, Germany analyzed with syngovia software (version VB60A, Hofix05). Two experienced neuroradiologists analyzed cerebral CT angiograms and digital subtraction angiograms (DSA) to evaluate the presence of different anatomical variants of CoW. The collateral-positive group consisted of patients with present PCoA and/or ACoA on the side of the occluded vessel. If the patient had ACoA, they was considered to be collateral-positive only if an ipsilateral A1 segment was present. If the patient had ACoA but aplasia of the A1 segment on the ipsilateral side, they was considered a collateral-negative. The collateral-negative group also included patients with neither communicating arteries at the side of the occluded vessel, as shown in Figure 1.

### 2.3. Endovascular Treatment

Mechanical thrombectomy was performed under local or general anesthesia with approved endovascular devices (aspiration catheters, stent retrievers, or a combination of both).

### 2.4. Main Outcomes and Measures

Early and post-intervention neurological outcomes (intracranial hemorrhage, subarachnoid hemorrhage, in-hospital death, and modified thrombolysis in cerebral infarction (mTICI) scale) were also collected and analyzed. Complete reperfusion was marked by an mTICI score of 3, while a successful reperfusion was defined by mTICI scores of 2B, 2C, and 3. The clinical efficacy outcome was the functional independence rate measured by mRS, which ranged from 0 to 6, with 0 meaning asymptomatic (no disability) and 6 meaning death. Successful functional recovery was considered an mRS score of 0–2 90 days after a stroke incident. We also analyzed the patient’s non-enhanced brain CT scans obtained 24 h after mechanical thrombectomy and scored the patients on the following scale: patients with a score of 0 had no infarction detected; a score of 1 included patients with a small or lacunar ischemic lesion; patients with a score of 2 had an ischemic lesion occupying less than 50% of the irrigation zone of the MCA; and a score of 3 included patients with a large infarct (more than 50% of the affected vessel area). Also, complications like intracerebral (ICH) and subarachnoid hemorrhage (SAH) were noted.

### 2.5. Statistical Analysis

Continuous variables are presented as median and interquartile range, whereas discrete variables are presented as percentages. Multivariate analysis was performed by using linear or logistic regression. Details of models are presented in the Supplementary Tables S1–S4. As a measure of statistical evidence, R^2^ or Tjur’s R^2^, evidence ratio (ER) based on difference in Akaiake Information criterion (ΔAIC) and *p* values were used. 95% confidence intervals were used as an uncertainty measure of the estimates, they were estimated by maximum likelihood. *p* values were interpreted according to the ASA statement on *p* values.

## 3. Results

This retrospective study included a total of 158 patients with acute ischemic strokes of the anterior circulation. There were 104 patients in the collateral-positive group, while the collateral-negative group included 54 patients. The median patients’ age did not differ among groups (76.5 vs. 78.5, *p* = 0.657). Other baseline characteristics of these two groups were also comparable, with no significant difference (Table 1).

In both groups, the most common site of occlusion was the M1 segment of the middle cerebral artery (73.1% of patients in the collateral-positive group and 63% in the collateral-negative group) (Table 2). Tandem occlusion was the rarest form of the LVO (1% in the collateral-positive group, 1.9% in the collateral-negative group). The aspiration catheter was the most common device used during MT; it was used in 67.3% of procedures in the collateral-positive group and in 68.5% of procedures in the collateral-negative group. The rates of unsuccessful mechanical thrombectomies due to the difficult anatomy of the aortic arch or extensive calcified plaques on the puncture site were similar in both groups (15.4% in the collateral positive group and 22.2% in the collateral negative group). Successful reperfusion (mTICI 2B, 2C, and 3) was accomplished in 71.1% of collateral-positive patients, with mTICI 3 in 60.6% of patients, and in 61.1% of patients in the collateral-negative group, with mTICI 3 in 50% of patients.

Hemorrhagic transformation was reported in both groups, but they did not differ in incidence. Systolic blood pressure above 140 mmHg did not affect the occurrence of ICH and SAH (OR: 1.871, 95% CI: 0.5252 to 7.271, *p* = 0.342). However, in patients with SBP below 140 mmHg, there was a trend toward a higher rate of successful functional outcome measured by mRS (OR: 0.2765, 95% CI: 0.08695 to 0.8176, *p* = 0.023).

The correlation of the presence of communicating arteries with radiological and clinical outcomes is shown in Table 3, the details of models regarding each of the outcomes can be seen in Supplementary Tables S1–S4.

The presence of just one communicating artery did not affect the NIHSS score on admission. If both communicating arteries were present, the NIHHS score was still unaffected (β= −1.584, 95% CI: −3.621 to 0.4531, *p* = 0.1259), but ACoP had a higher impact on lowering its score (β= −1.875, 95% CI: −3.431 to −0.3188, *p* = 0.019).

Odds for successful recanalization after mechanical thrombectomy seem to be associated with the presence of primary collaterals (OR: 6.844; 95% CI: 1.278 to 52.21 *p* = 0.036) and the total number of collaterals (OR: 4.696; 95% CI = 1.381 to 26.92; *p* = 0.036) (Table 3). The presence of communicating arteries was associated with a lower score of ischemic lesion on follow-up non-enhanced brain CT (β = −0.3520, 95% CI −0.6569 to −0.04699, *p* = 0.024) (Table 3). The presence of both the anterior and posterior communicating artery did not contribute to additional lowering of the score of ischemic stroke on control CT (β= −0.2551, 95% CI: −0.6632 to 0.1530, *p* = 0.218), but a present ACoA was more important than AcoP in lowering the mentioned score (β= −0.3339, 95% CI: −0.6382 to −0.0295, *p* = 0.031).

We also collected information on patients’ recovery three months after the onset of stroke. In the collateral-positive group, we obtained mRS scores for 92/104 (88.5%) patients, and good recovery (mRS score 0–2) was found in 53.3% of patients, while 26.1% of patients died. The causes of death in ten out of twenty-four patients were pneumonia (four patients), urinary infection (one patient), cardiac decompensation (three patients), and two patients were already in treatment for malignancy. In the collateral-negative group, we obtained mRS scores of 52/54 (96.3%) patients. Successful functional recovery was confirmed in 28.8% of patients and death in 48.1% of patients. Out of the twenty-five patients who died, four had cardiac failure, three were treated for pneumonia, and one died of pulmonary embolism during their hospital stay.

The odds for favorable functional recovery measured with the mRS score were 11.87 times higher in patients who had AcoA or PcoA than in the collateral-negative group (95% CI 2.952 to 61.60, *p* = 0.001) (Table 3). Moreover, the presence of both communicating arteries increased the odds of achieving a good functional outcome even more (OR: 29.13, 3.875–377.8, *p* = 0.003), with ACoA having a greater impact on achieving a better recovery after stroke than ACoP (OR: 12.62, 2.691–85.18, *p* = 0.003).

The presence of collaterals seems to be the only factor associated with favorable functional outcomes in patients who had a successful recanalization score after mechanical thrombectomy (Table 4).

## 4. Discussion

Our study evaluated the anatomy of CoW and its impact on recanalization and functional outcomes after stroke. It showed that patients with AIS and present primary collaterals had a higher rate of functional independence after LVO treated with EVT.

Several studies have already investigated anatomical variants of CoW and their effect on outcomes after ischemic stroke, but the results have been conflicting. Westphal et al. [20] classified CoW variants into four vascular models, taking into account both ipsilateral and contralateral A1, the presence of ACoA, the fetal variant of PCA, and ipsilateral PCoA or P1 segments using TOF-MRA. The correlation of CoW variants on clinical outcomes in patients with LVO stroke treated with EVT was not found [20], which was confirmed by others [21,22]. Opposite of that, some reports did find that incomplete CoW may be associated with a less favorable outcome for stroke patients [23,24,25,26].

Variants in posterior circulation occurred more often in patients with stroke [23,24,25,26] and were detected with various radiological imaging methods. Some studies based their classification on measuring vessel diameter, while others analyzed only vessels of anterior or posterior circulation due to technical reasons [23]. A study by Zhou et al. [26] used MRA and found that NIHSS scores at admission and discharge were significantly higher in the incomplete CoW group than in the complete CoW group, alongside better mRS scores in patients with a complete circle of Willis.

One of the reasons for these conflicting results among published papers may be different classifications of anatomical variants or the small number of patients included in the study. Some authors proposed two or more vascular model groups, focusing primarily on whether the CoW was completely closed [23].

A number of studies analyzed collateral circulation, but they were based on the angiographic evaluation of peripheral, i.e., secondary collaterals. They used different scoring systems depending on which radiological imaging was used (ASPECTS, CT-SI, or TAN) [27,28].

A study by Song et al. [29] evaluated the Alberta Stroke Program Early CT Score (ASPECTS) [29] and CT angiography (CTA-CS) for grading collateral circulation in patients with AIS of anterior circulation. They reported that the predictability of successful reperfusion improved when using both scoring systems. Higher scores were associated with favorable outcomes. An extensive study by Liebeskind et al. [30] evaluated collateral circulation in cerebral angiography, and grades were assessed in accordance with the American Society of Interventional and Therapeutic Neuroradiology/Society of Interventional Radiology (ASITN/SIR) score [31], which is considered a gold standard for the evaluation of collaterals on DSA. The authors concluded that well-developed collaterals were associated with better recanalization, reperfusion, and subsequent clinical outcomes. Additional studies came to the same conclusions [32,33,34,35,36,37,38,39] and found that successful reperfusion was a strong predictor of favorable outcomes in both good and poor collateral groups [39].

Additional analysis showed that collaterals had a direct impact on the percentage of penumbra that was salvageable [40,41], with the important conclusion that the penumbra is maintained only for a certain period of time (thus extending the time frame for EVT). Even “late time window” patients with good collateral grade treated with MT express smaller penumbra and better functional outcomes [42]. Other authors, though, argue that collateral status is associated only with core volume and its growth speed [33,34,35,36,41,42,43] and that only reperfusion success, and not collateral status, is an independent predictor of functional outcome at discharge [44]. However, patients with poor collateral possibilities can also benefit from MT in the early time window of LVO, which results in a more substantial limitation of infarct growth [45].

We evaluated blood pressure at admission since it is a predictor of a less favorable functional outcome after MT [46,47,48] and is also related to the risk of procedural complications such as subarachnoid and intracerebral hemorrhage [49,50,51]. Our results were not in accordance with these studies, probably due to the selected cut-off value of SBP (140 mmHg). The authors of a recent study recommended a reduction in blood pressure within 24 h after admission, even in stroke patients without recanalization therapy, as it had a positive effect on the clinical prognosis [52]. It is reported that patients with poor collaterals have a higher frequency of hemorrhagic transformation, both symptomatic and asymptomatic [35,53], which was not proven in our study.

All the aforementioned studies indicate that collateral circulation plays an important role in the pathophysiology of stroke. It slows tissue deterioration from penumbra to infarction, extends the therapeutic time window, and reduces the final stroke volume, with important implications for clinical outcomes [9]. A study by Baron et al. [54] even suggests that collateral circulation status should be considered alongside symptom onset time when making therapeutic decisions. It is a task for future studies to find the most suitable classification and radiological modality for assessing collateral circulation, leading to a better selection of patients who will benefit the most from endovascular therapy.

One of the limitations of our study was the relatively small number of patients included. However, this is due to strict inclusion and exclusion criteria. Furthermore, our study was detailed in the analysis of numerous factors affecting outcomes after stroke, such as preexisting comorbidities and further detailed follow-up of the treated patients. Another advantage of our study was the inclusion of DSA analysis, which confirmed CTA findings, while other studies with CoW were based entirely on TOF-MRA or CTA, which are susceptible to artifacts.

## 5. Conclusions

“Time is brain” is a well-known paradigm when talking about stroke, which means the reperfusion should be delivered as soon as possible. Our study confirmed that the selection of patients for endovascular treatment should take into account all risk factors for stroke, as well as factors predicting favorable outcomes after the treatment, like the status of collaterals at initial CTA.

## Figures and Tables

**Table 1:** Baseline characteristics.

	Collateral-Positive Group (*n* = 104)	Collateral-Negative Group (*n* = 54)	*p* Value
Age, years (median, IQR)	78.5 (71–83)	76.5 (67–84)	0.657 *
Women (no, %)	65 (62.5%)	33 (61.1%)	0.865 ^†^
Arterial hypertension (no, %)	71 (68.3%)	39 (72.2%)	0.710 ^†^
Diabetes (no, %)	24 (23%)	11 (20.3%)	0.839 ^†^
Atrial fibrillation (no, %)	51 (49%)	33 (61.1%)	0.169 ^†^
Antiplatelet/anticoagulation therapy (no, %)	34 (32.7%)	26 (48.1%)	0.082 ^†^
Baseline NIHSS (median, IQR)	15 (14–17)	15 (11.5–17)	0.397 ^†^
Systolic blood pressure (median, IQR)	140 (130–170)	145 (120–162.5)	0.576 *
Diastolic blood pressure (median, IQR)	80 (70–95)	80 (70–90)	0.406 *
IVT	25/38 (65.8%)	13/19 (68.4%)	0.844 ^†^
Hemorrhage on control CT	21 (20.2%)	15 (27.7%)	0.322 ^†^

* Mann–Whitney test^; †^ Fischer’s exact test; NIHSS = The National Institutes of Health Stroke Scale; IVT = intravenous thrombolysis.

**Table 2:** Stroke outcomes.

	Collateral-Positive Group (*n* = 104)	Collateral-Negative Group (*n* = 54)
Localisation of thrombus		
M1 segment	76/104 (73.1%)	34/54 (63%)
M2 segment	12/104 (11.5%)	3/54 (5.5%)
T-occlusion	15/104 (14.4%)	16/54 (29.6%)
tandem occlusion	1/104 (1%)	1/54 (1.9%)
EVT technique		
aspiration only	70/104 (67.3%)	37/54 (68.5%)
aspiration and stent retriever	18/104 (17.3%)	5/54 (9.3%)
unsuccessful	16/104 (15.4%)	12/54 (22.2%)
Good reperfusion (TICI 2B, 2C or 3)	74/104 (71.1%)	33/54 (61.1%)
TICI 3 (%)	63/104 (60.6%)	27/54 (50%)
Ischemic lesion on control CT		
0	14/103 (13.6%)	6/54 (11.1%)
1	46/103 (44.7%)	15/54 (27.8%)
2	29/103 (28.1%)	17/54 (31.5%)
3	14/103 (13.6%)	16/54 (29.6%)
Hemorrhage on control CT		
SAH	10/20 (50%)	6/15 (40%)
ICH	10/20 (50%)	9/15 (60%)
Modified Rankin Scale Score		
0–2	49/92 (53.3%)	15/52 (28.8%)
3–5	19/92 (20.6%)	12/52 (23.1%)
6	24/92 (26.1%)	25/52 (48.1%)

EVT = Endovascular therapy; SAH = subarachnoid hemorrhage; ICH = intracerebral hemorrhage.

**Table 3:** Prediction of outcome factors in case of presence of communicating arteries.

Outcomes	OR or β	95% CI	*p* Value
NIHSS score	−1.164	−2.784 to 0.4555	0.1567
Successful recanalization (TICI score 2B, 2C, 3)	6.844	1.278 to 52.21	0.036
Control CT score	−0.3520	−0.6569 to −0.04699	0.024
Favorable functional recovery (mRS 0–2)	11.87	2.952 to 61.60	0.001

NIHSS = The National Institutes of Health Stroke Scale; TICI = thrombolysis in cerebral infarction (TICI) scale; mRS = Modified Rankin Scale for Neurologic Disability.

**Table 4:** Multivariate analysis of variables associated with favorable functional recovery (mRS 0–2) in patients with successful recanalization score on mechanical thrombectomy (TICI 2B-3).

Variable	OR	95% CI	*p* Value
Age	0.9436	0.8494 to 1.028	0.2211
Sex	0.6433	0.1344 to 2.908	0.5668
Present collaterals	2.617	1.047 to 7.521	0.0505
Antiplatelet therapy	0.7220	0.1486 to 3.512	0.6800
NIHSS score	0.9537	0.7739 to 1.178	0.6498
SAH or ICH	0.4691	0.08010 to 2.373	0.3697

NIHSS = The National Institutes of Health Stroke Scale; SAH = subarachnoid hemorrhage; ICH = intracerebral hemorrhage.

## Data Availability

Data are contained within the article and supplementary materials.

## Supplementary Materials

The following supporting information can be downloaded at: https://www.mdpi.com/article/10.3390/biomedicines11113008/s1.
