# Supplementary material for: The Presence of Communicating Arteries in the Circle of Willis Is Associated with Higher Rate of Functional Recovery after Anterior Circulation Ischemic Stroke
Source: Biomedicines. 2023 Nov 9;11(11):3008. doi: 10.3390/biomedicines11113008 (PMC10669712; doi:10.3390/biomedicines11113008)
Supplement: Supplementary file 1 [file biomedicines-11-03008-s001.zip › biomedicines-2690349-supplementary.pdf]

Supplementary Table S1. Correlation of presence of communicating arteries with NIHSS. A model without communicating arteries (R^2=1.1%) as variable is 1.094 (ΔAIC=-0.18) times more likely than the model that has presence of communicating arteries as variable (R^2=3.3%).

Covariate	*β*	95% CI (asymptotic)	P value
Intercept	13.72	8.044 to 19.40	<0.0001
age	0.01513	-0.06382 to 0.09407	0.7043
female	0.3415	-1.386 to 2.069	0.6955
Antiaggregating Tx.	0.6820	-0.9615 to 2.326	0.4118
# of comorbidities	-0.2573	-1.268 to 0.7539	0.6144
presence of comm. arteries	-1.164	-2.784 to 0.4555	0.1567

Supplementary Table S2. Correlation of presence of communicating arteries with successful recanalization. A model without communicating arteries (Tjur's R^2=0.2943) as variable is 3.7 (ΔAIC=-2.6) times more likely than the model that has presence of communicating arteries as variable (Tjur's R^2=0.2387).

Variable	OR	95% CI (profile likelihood)	P value
Intercept	256758	64.20 to 41940005616	0.0130
age	0.8957	0.7658 to 0.9934	0.0870
female	0.7908	0.08925 to 5.185	0.8130
Antiaggregating Tx.	5.287	0.9196 to 49.42	0.0903
# of comorbidities	2.500	0.8210 to 8.685	0.1202
presence of comm. arteries	6.844	1.278 to 52.21	0.0359
Aspiration wo. stent* vs. with stent	0.09418	0.009861 to 0.5944	0.0196
NIHSS	0.8973	0.6718 to 1.171	0.4336

Supplementary Table S3. Correlation of presence of communicating arteries with ischemic lesion on control CT (score from 0 to 3). A model without communicating arteries (R^2=15.28%) as variable is 4.795 (ΔAIC=-3.315) times more likely than the model that has presence of communicating arteries as variable (R^2=12.26%).

Variable	*β*	95% CI (asymptotic)	P value
Intercept	2.523	1.495 to 3.550	<0.0001
age	-0.005848	-0.01931 to 0.007613	0.3920
female	0.02265	-0.2917 to 0.3370	0.8870
Antiaggregating Tx.	0.2126	-0.1077 to 0.5329	0.1917
# of comorbidities	0.1183	-0.06612 to 0.3027	0.2069
comm. arteries	-0.3520	-0.6569 to -0.04699	0.0240
Aspiration wo. stent	-0.8366	-1.222 to -0.4513	<0.0001
Aspiration with stent	-0.4825	-1.009 to 0.04394	0.0721

Supplementary Table S4. Correlation of presence of communicating arteries with Favorable functional recovery (mRS 0-2). A model without communicating arteries (Tjur's R^2=0.2943) as variable

is 3.7 (ΔAIC=-2.6) times more likely than the model that has presence of communicating arteries as variable (Tjur's R^2=0.2387).

Variable	OR	95% CI (profile likelihood)	P value
Intercept	229.0	0.6501 to 114636	0.0716
age	0.8998	0.8183 to 0.9687	0.0123
female	0.7574	0.1747 to 3.221	0.7035
Antiaggregating Tx.	1.618	0.3561 to 7.929	0.5357
# of comorbidities	0.5309	0.2004 to 1.344	0.1827
presence of comm. arteries	11.87	2.952 to 61.60	0.0012
Aspiration wo. stent	67.63	6.238 to 2489	0.0041
Aspiration with stent	8.834	0.5299 to 351.4	0.1682
NIHSS	0.8992	0.7371 to 1.081	0.2679
